# Longitudinal Spinous‐Splitting Laminoplasty with Coral Bone for the Treatment of Cervical Adjacent Segment Degenerative Disease: A 5‐Year Follow‐up Study

**DOI:** 10.1111/os.13027

**Published:** 2021-12-22

**Authors:** Wei He, Da He, Qi‐long Wang, Wei Tian, Bo Liu, Ya‐jun Liu, Yu‐qing Sun, Yong‐gang Xing, Ning Yuan, Qiang Yuan, Bin Xiao, Bing Han, Yu‐mei Wang, Teng‐fei Ma, Ming‐ming Liu

**Affiliations:** ^1^ Department of Spinal Surgery Beijing Jishuitan Hospital Beijing China

**Keywords:** Adjacent segment disease, Cervical vertebrae, Laminectomy, Spinous process

## Abstract

This study was designed to analyze the causes of cervical adjacent segment degenerative disease (ASDis), evaluate the surgical outcomes of longitudinal spinous‐splitting laminoplasty with coral bone (SLAC) during cervical reoperation, and accumulate data on reoperation with SLAC in a primary hospital. Based on the inclusion and exclusion criteria, we conducted a retrospective study involving 52 patients who underwent cervical reoperation for ASDis using SLAC at the spinal surgery department of the Beijing Jishuitan Hospital from 1998 to 2014. Among them, 39 were treated with anterior cervical fusion and internal fixation during the first operation (anterior cervical corpectomy with fusion [ACCF], *n* = 24; anterior cervical discectomy and fusion [ACDF], *n* = 11; and cervical disc arthroplasty [CDA], *n* = 4). Outcomes were the Japanese Orthopaedic Association (JOA) score, neck disability index (NDI) score, upper limb/neck and shoulder evaluated using a visual analogue scale (VAS), and rates of ASDis. In patients who underwent an anterior cervical approach in the first instance, the incidence of ASDis was significantly higher in the C_3/4_ gap than in the other gaps. In the ACCF group, the lateral radiograph of the cervical spine revealed that the distance between the anterior cervical plate and the adjacent segment disc was <5 mm in 15 (62.5%) cases and five (12.8%) cases, respectively, the internal fixation screws broke into the annulus of the adjacent segment. After the first SLAC, ASDis developed at C_2/3_ and C_3/4_ in four (30.8%) and eight (61.5%) cases, respectively. After reoperation, all cases were followed up for >5 (average, 6.2) years. The pre‐reoperation and last follow‐up values were as follows: mean Japanese Orthopaedic Association score, 10.2 ± 1.5 *vs* 15.5 ± 0.7 (*P* = 0.03); neck disability index, 26.2 *vs* 13.6 points (*P* = 0.01); upper‐limb visual analog scale (VAS) score, 6.1 *vs* 2.6 points (*P* = 0.04); and neck and shoulder VAS score, 6.6 *vs* 2.1 points (*P* = 0.03). SLAC is a simple technique in which the local anatomy is clearly visible and satisfactory clinical outcomes are obtained.

## Graphical abstract

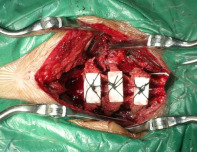

This study was designed to evaluate the surgical outcomes of longitudinal spinous‐splitting laminoplasty with coral bone (SLAC) during cervical reoperation. SLAC is a simple technique in which the local anatomy is clearly visible and satisfactory clinical outcomes are obtained.

## Introduction

Cervical surgery has been widely used in the treatment of cervical trauma, tumors, deformity, and degenerative diseases^1^, ^2^, ^3^, ^4^, ^5^, ^6^, ^7^. The evaluation of cervical spine surgery is based on the medical knowledge of the disease at the time, the developmental status of the internal fixation device, and the technical skill of the surgeon. However, with the rapid development of new theories and new techniques in the field of cervical spine surgery, there is a greater likelihood that spine surgeons will make poor choices and technical errors if they are not aware of the various surgical techniques applied to the cervical vertebrae or if they are biased in their use of techniques.

**Fig. 1:**
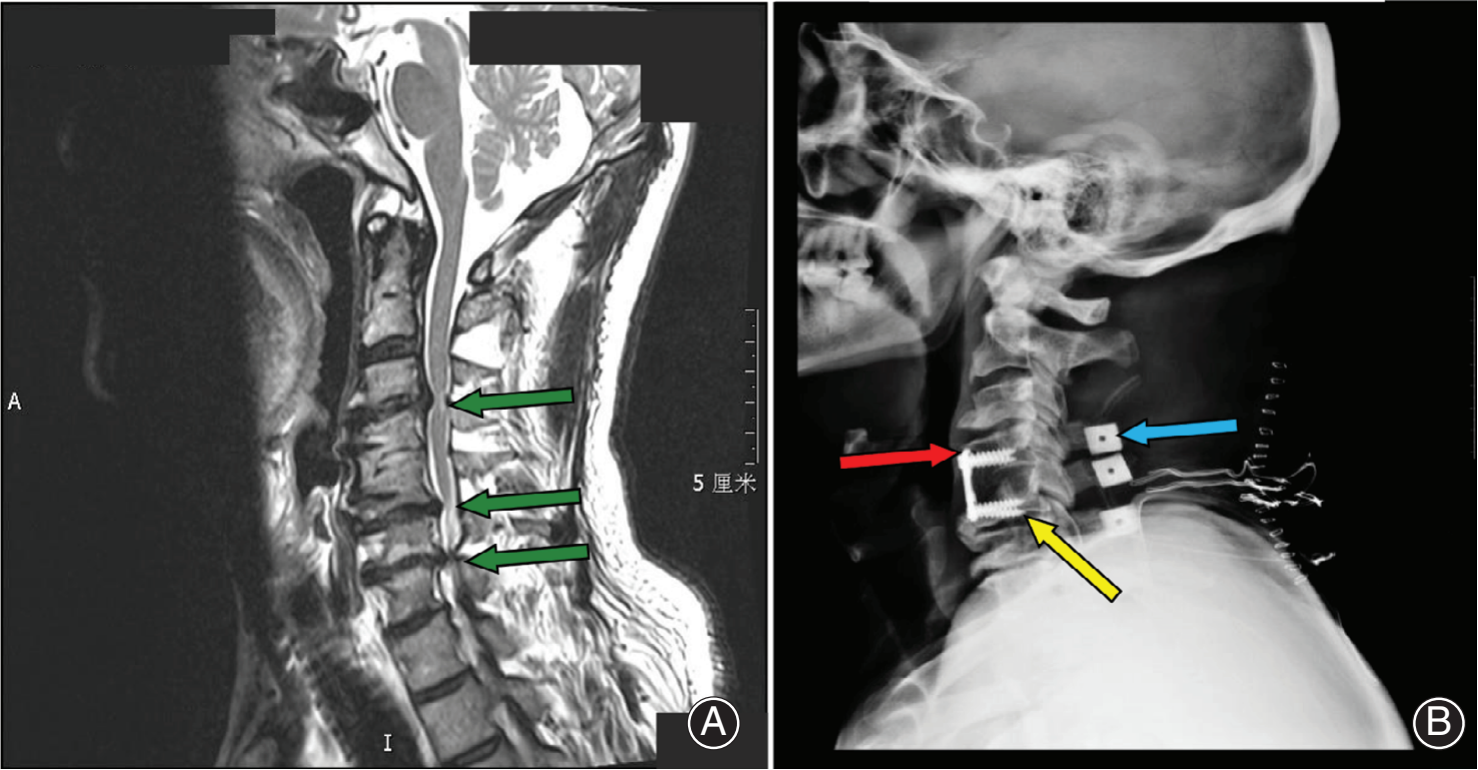
Preoperative and postoperative radiological imaging scans of a patient. (A) The first postoperative MRI imagine. Cervical MR scan obtained after the first C_4/5_ ACDF. Postoperative scan showing ASDis at C_3/4_ and C_5/6_‐disc herniation, corresponding to the flat spinal cord compression (green arrow). (B) The second postoperative X‐Ray imagine. Radiograph obtained after the reoperation (SLAC). The distance between the vertebral plate and the lower edge of the C_3/4_ intervertebral space is <5 mm (red arrow). The C_5_ vertebral body screw has broken through the C_5_ vertebral endplate and the C_5/6_ annulus (yellow arrow). The figure shows that there is an artificial bone (blue arrow) between the C_4_, C_5_, and C_6_ spinous processes.

**Fig. 2:**
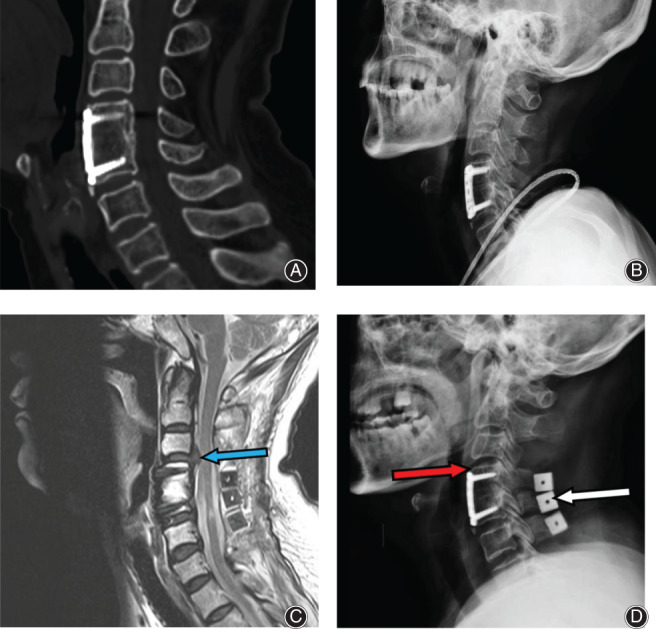
Preoperative and postoperative radiological imaging scans of a patient. (A) The first postoperative CT imagine. (B) The first postoperative X Ray imagine. (C) The second postoperative cervical MR scan. ASDis develops at C_3/4_; the disc protrudes to the spinal canal (blue arrow), and the spinal cord is compressed. (D) The second postoperative X‐ray imagine. The distance between the vertebral plate and the lower edge of the C_3/4_ intervertebral space is <5 mm (red arrow). Lateral cervical radiograph obtained after reoperation with SLAC: an artificial bone (white arrow) is present between the C_4_, C_5_, and C_6_ spinous processes. MR, magnetic resonance; ACDF, anterior cervical discectomy and fusion; ASDis, adjacent segment degenerative disease; SLAC, spinous‐splitting laminoplasty with coral bone.

In recent years, adjacent segment disease (ASD), which develops after cervical spine surgery, has gained attention as an issue in the long‐term care of patients^8^, ^9^, ^10^, ^11^. Adjacent segment degeneration (ASDeg; Figs 1A,2A) is the simple degeneration of adjacent segments and includes disc degeneration, osteophyte formation, articular process hyperplasia, and spinal stenosis without clinical symptoms. Adjacent segment degenerative disease (ASDis) refers to a new radiculopathy or myelopathy at adjacent segments.

The therapeutic goal of ASDis treatment is to resolve the new spinal cord/neural root compression in the adjacent segment degeneration and correct new malformations or instability, taking into account the stability of the reconstructed spine. The difficulty and risks of the second operation are much greater than those of the first. In addition, factors such as distrust of doctors may complicate communication between doctors and patients regarding reoperation. Moreover, patients' unrealistic expectations of surgery, the anxiety of patients and their families, and the financial struggles of families may also have an influence.

In this study, we summarize the cases of cervical spine reoperation due to ASDis noted in our hospital between January 1998 and January 2014. In appropriate cases, longitudinal spinous‐splitting laminoplasty with coral bone (SLAC) was performed. This study aimed to evaluate the surgical results of SLAC and compile our experience regarding reoperation with SLAC. Our research questions included the following. First, are patients with ASDis undergoing SLAC procedure feasible and safe? Second, does SLAC result in satisfactory clinical outcomes? Third, what perioperative complications are associated with SLAC?

## Methods

### Study Population

**Fig. 3:**
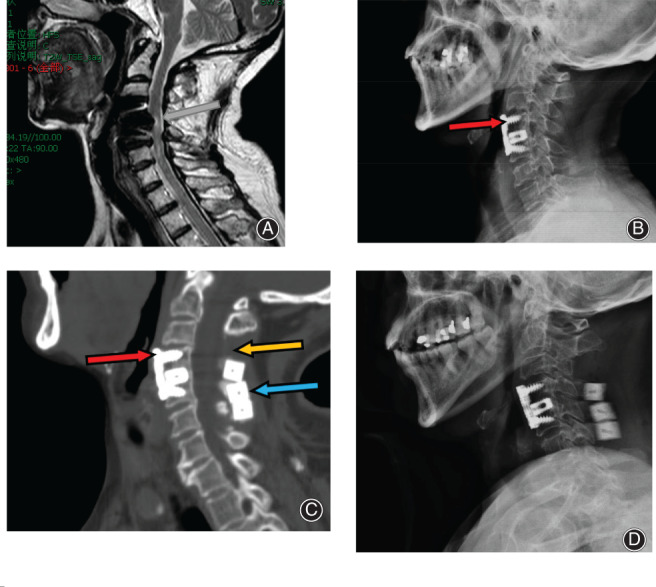
Preoperative and postoperative radiological imaging scans of a patient. (A) The first postoperative MR imagine. The dural sac in the spinal canal at the same level of the C_4‐5_ vertebral body was compressed. (B) The first postoperative X‐ray imagine. The distance between the vertebral plate and the lower edge of the C_3/4_ intervertebral space is <5 mm (red arrow). (C) The second postoperative CT imagine. Images obtained after reoperation with SLAC: postoperative cervical CT scan showing that the distance between the vertebrae plate and the lower edge of the C_3/4_ intervertebral space is <5 mm (red arrow). Image obtained during C_3_ laminectomy (orange arrow). An artificial bone (blue arrow) is visible between the C_4_, C_5_, and C_6_ spinous processes. (D) The second postoperative X‐ray imagine.

The inclusion criteria were as follows: (i) patients with worsening postoperative neurological symptoms, or new spinal cord compression, or nerve root symptoms; (ii) SLAC for the second surgery; (iii) Self‐comparison of the patient's clinical symptoms before and after the second operation; (iv) Japan Orthopaedic Society (JOA) score, neck disability index (NDI) score, and visual analog scale (VAS) score (upper limb/neck and shoulder); Ccervical vertebrae lateral radiograph, which was defined as the distance between the anterior cervical plate and the adjacent segment at <5 mm (Figs 1B,2B,3B), and the internal fixation screw breaking through the adjacent segmental annulus (Fig. 1B); and (v) retrospective analysis. The exclusion criteria were: (i) unstable cervical vertebrae, (ii) internal fixation failure, (iii) non‐union of the bone graft, (iv) cervical kyphosis deformity >10°, and (v) intraspinal pressure of the adjacent segment accounting for >50% of the spinal canal cross‐section.

The study was approved by the Ethics Committee of Beijing Jishuitan Hospital (approval number: 201905), and informed consent was waived due to the retrospective design of the study.

### Surgical Procedure

#### SLAC (C_2‐7_)

**Fig. 4:**
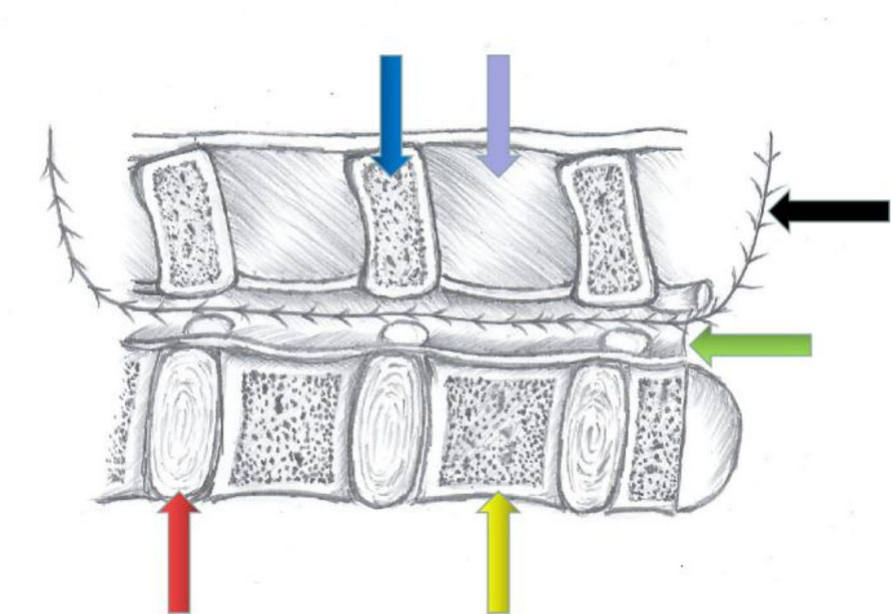
Schematic diagram of surgical operation, mid‐sagittal position of lumbar spine. A catheter and T‐saw were placed under the lamina, and the spinous processes of C_4_‐C_6_ were sawed with the T‐saw. Intervertebral disc (red arrow), vertebral body (orange arrow), spinous process (blue arrow), interspinous ligament (purple arrow), spinal canal (green arrow).

**Fig. 5:**
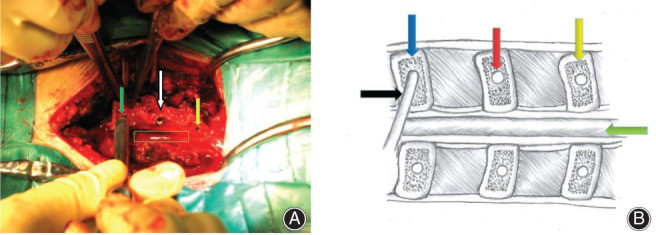
Holes are drilled in the spinous process (C_4‐6_). (A) Actual image of the operation. The white arrow shows the hole in the left half of the C_5_ processes. The yellow arrow shows the hole in the left half of the C_4_ processes. The green arrow shows the hole in the left half of the C_6_ processes (the hole has been drilled using a grinding drill). The rectangle with the green border shows the spinal cord in the C_4_‐C_6_ range. (B) Schematic diagram of surgical operation. The red arrow shows the hole in the left half of the C_5_ processes.The yellow arrow shows the hole in the left half of the C_4_ processes. The blue arrow shows the hole in the left half of the C_6_ processes (the hole has been drilled using a grinding drill–black arrow). The green arrow shows spinal canal.

**Fig. 6:**
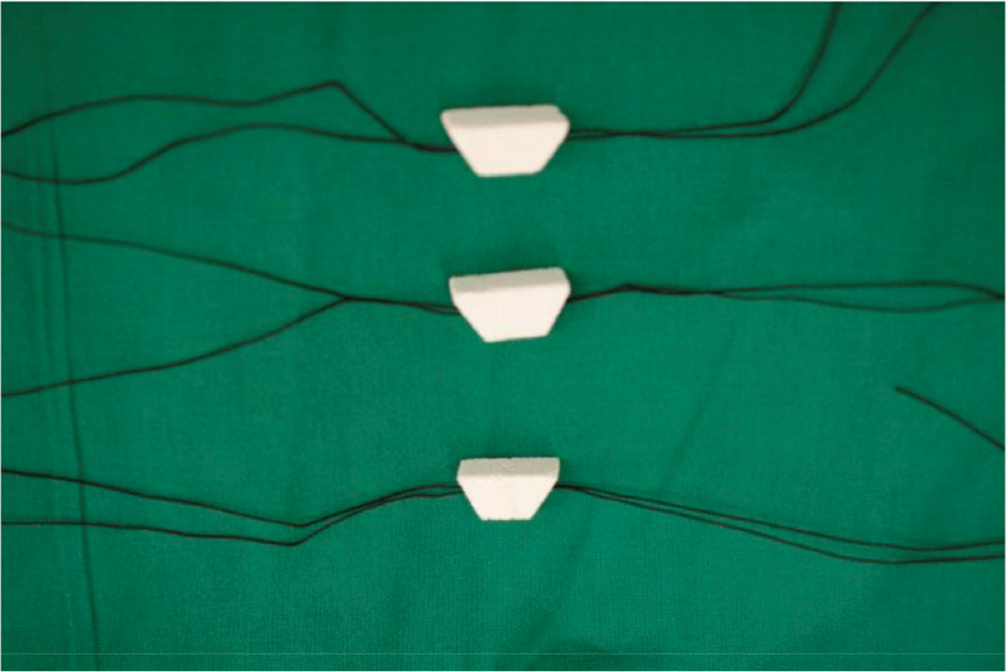
CHA (coralline hydroxyapatite) implant (10 mm× 20  mm× 10  mm× 10 mm) with two surgical suture silk thread.

**Fig. 7:**
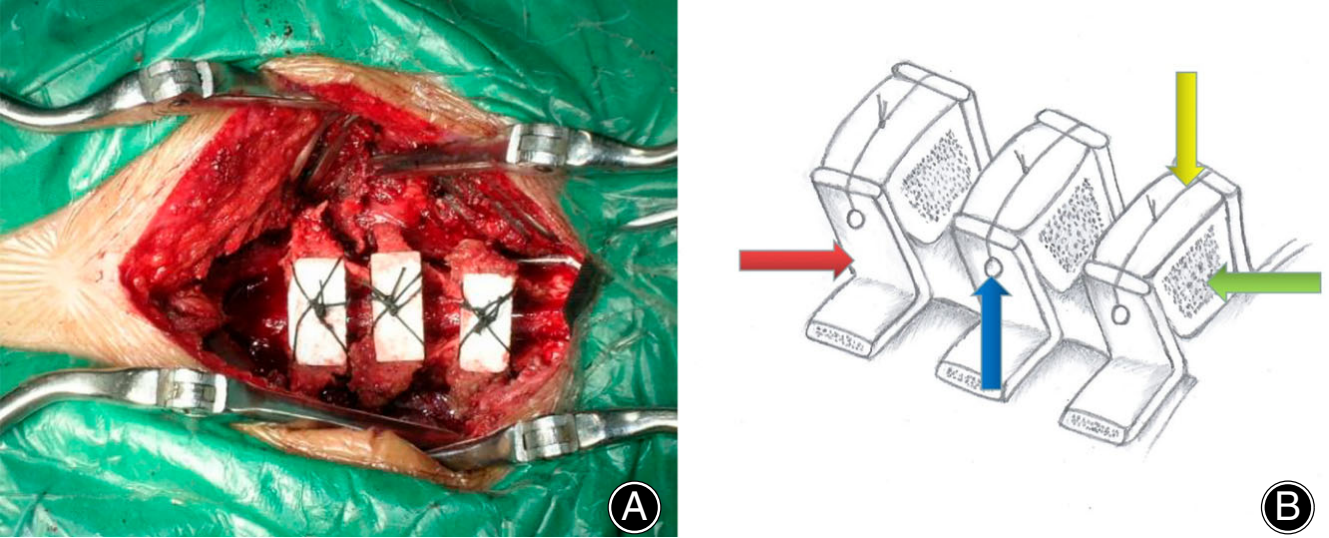
Postoperative effect of CHA implant were fixed in spinous process. (A) Actual image of the operation. CHA (coralline hydroxyapatite) implants are fixed between the left and right sides of the C_4_‐C_6_ spinous processes (with a width of about 2 cm). The blue arrow shows the right half of the C_5_ processes. The yellow arrow shows the surgical suture silk thread. (B) Schematic diagram of surgical operation. The red arrow shows the right half of the C_6_ processes.The blue arrow shows the hole in the right half of the C_5_ processes.The yellow arrow shows the surgical suture silk thread. The green arrow shows CHA implants.

1. After successful anesthesia, the patient was placed in the prone position so that the neck was in a slight flexion and the hands were on either side of the body.
2. A midline incision was made after C_2_‐C_7_, the skin and the soft tissue of the neck were cut subcutaneously, and the spinous process of C_3_‐C_7_ was exposed. Cobb's periosteal stripper technique was used to push the paravertebral muscles away from the periosteum. The surgical hemostasis device was then used to stop the bleeding. The bilateral rotator muscles were cut on the spinous process with scissors, carefully protecting the cervical semi‐spine muscles in C_2_ and C_7_. This exposed the C_3_‐C_7_ lamina.
3. The C_3_ and C_7_ lamina and C_2_ ventral lamina were removed with a grinding drill, an ultrasonic bone knife, and a laminar rongeur. The ligamentum flavum between C_6_ and C_7_ was removed to expose the underlying dura mater. A catheter and T‐saw were placed under the lamina, and the spinous processes of C_4_‐C_6_ were sawed with the T‐saw (Fig. 4).
4. A hinged structure, like that of a door shaft, was made with a grinding drill on the inner side of the small C_4_‐C_6_ joints on each side, until the depth of the contralateral cortex and the ventral cortex was preserved. The lamina was opened with tissue scissors and a small curette. The two sides were separated, and the ligamentum flavum was cut longitudinally to expose the underlying dura mater. The dura mater and pulsate were carefully observed.
5. Holes were drilled in the spinous process (Fig. 5A,B); the three coralline hydroxyapatite (CHA) implants (10 mm× 20 mm× 10 mm× 10 mm; Beijing YHJ Science and Trade Co., Ltd., Beijing, China; Fig. 6) were fixed between the left and right sides of the C4‐C6 spinous processes (with a width of about 2 cm) with two 10‐lines (Fig. 7A,B).
6. The wound was washed with saline, and a negative pressure drainage tube was placed in the wound after the bleeding was stopped completely. The wound was sutured layer‐by‐layer, and the operation was completed.
7. At this point, the operation was deemed successful, and the patient returned to the ward after surgery.

## Outcomes

### Japanese Orthopaedic Association (JOA)

The JOA score is composed of four sections: subjective symptoms (low back pain, leg pain, and gait), clinical signs (straight‐leg‐raising test, sensory and motor disturbances), restriction of activities of daily living (seven items), and urinary bladder function as minus points. The score can range from −6 (maximum disability) to 29 (no disability).

### Neck Disability Index (NDI) Score

The NDI assesses the functional status of adults with neck‐related pain. The NDI contains 10 functional status‐related questions scored from zero (no pain) to five (most severe pain). Scores are expressed as a percentage of total points, with lower scores representing better functioning and less disability.

### Upper Limb/Neck and Shoulder

The visual analogue scale (VAS) of the upper limb/neck and shoulder were analyzed to evaluate the effects of treatment. Using a VAS ruler, the score was determined by measuring the distance (cm) on the 10‐cm line between the “no pain” anchor and the patient's mark, providing a range of scores from 0 to 10. A higher score indicated greater pain intensity. Patients described their pain intensity as 0 (no pain) to 10 (worst pain ever).

#### Statistical Analyses

Statistical analysis was performed using SPSS 18.0 for Windows (IBM, Armonk, NY, USA). Data were analyzed using the paired t‐test and represented as mean ± standard deviation. A value of *P* < 0.05 was considered statistically significant.

## Results

### Patient Information

This study included 52 patients (37 men and 15 women). The median age of the patients was 58.3 years (47–78 years). For the first procedure (Table 1), 11 patients underwent anterior cervical discectomy and fusion (ACDF), 24 underwent anterior cervical corpectomy with fusion (ACCF), and four underwent cervical disc arthroplasty (CDA). The median interval between the first surgery and reoperation was 74 months (7–242 months). Thirteen patients underwent SLAC for the first surgery. The median interval between the first surgery and reoperation was 33 months (21–59 months). Before reoperation, 31 patients suffered from neck and shoulder pain, 11 patients had upper limb pains/numbness/inability, and 10 patients had spinal cord compression symptoms.

**TABLE 1:** Preoperative data of patients undergoing operation with anterior cervical surgery for the first time

	Total	ASDis section C_3/4_	Steel plate from adjacent segments <5 mm	Internal fixation screw breaks through adjacent fiber ring	Compressed tissue accounts for >50% of spinal canal area
ACCF	24	16 (66.7%)	15 (62.5%)	3 (12.5%)	0
ACDF	11	5 (45.5%)	0	2 (18.2%)	0
CDA	4	2 (50.0%)	0	0	0
Total	39	23 (58.9%)		5 (12.8%)	0

ACCF, anterior cervical corpectomy with fusion; ACDF, anterior cervical discectomy and fusion; ASDis, adjacent segment degenerative disease; CDA, cervical disc arthroplasty.

### Rates of ASDis

After the first anterior cervical surgery, rates of ASDis were significantly higher in C_3/4_ than in other gaps (66.7% in the ACCF group, 45.5% in the ACDF group, and 50.0% in the CDA group). In the ACCF group, 62.5% cases were identified based on the lateral radiograph of the cervical spine in which the distance between the anterior cervical plate and the adjacent segment was <5 mm. In five (12.8%) cases the internal fixation screws broke into the annulus of the adjacent segment.

There were 13 cases of first‐time SLAC, including four (30.8%) cases in which ASDis developed at C_2/3_ and eight (61.5%) cases in which ASDis developed at C_3/4_.

After reoperation, all cases were followed‐up for >5 years, with an average follow‐up period of 6.2 years. Nerve damage or internal fixation failure were not reported.

### JOA, NDI, and VAS

The mean JOA score of the 52 patients was 10.2 ± 1.5 before the reoperation and 15.5 ± 0.7 at the last follow‐up. There was a significant difference between the preoperative score and the follow‐up score (*P* = 0.03). The NDI score was significantly higher before the reoperation (26.2 points) compared to that at the last follow‐up (13.6 points; *P* = 0.01). The upper limb VAS score was significantly higher (6.1 points) before reoperation compared to that at the last follow‐up (2.6 points; *P* = 0.04). The neck and shoulder VAS score was significantly higher (6.6 points) before reoperation compared to that at the last follow‐up (2.1 points; *P* = 0.03).

## Discussion

### C_3_ _/4_ is Prone to ASDis

After the first anterior cervical surgery, ASDis was significantly higher in C_3/4_ than in other gaps. Our results suggest that ASDis is more likely to develop at C_3/4_. Furthermore, among the 13 cases of first‐time SLAC, ASDis developed at C_3/4_ in eight (61.5%) cases.

Yue *et al*.^12^ and Ishihara *et al*.^13^ found that ASDeg increased at a rate of 3%–8% per year after 10 years of follow‐up after ACDF. Hashimoto *et al*.^10^ reported that after cervical fusion, the incidence of imaging ASDeg was 32.8%, and 1/4 to 1/3 of cases eventually developed into clinically symptomatic ASD. Hilibrand *et al*.^14^ showed that among patients undergoing ACDF, 25.6% develop ASDeg within 10 years after surgery. Hilibrand *et al*.^11^ reported a 2.9% rate of reoperation in patients with ASDis development and no symptomatic improvement after receiving conservative treatment; however, other scholars believe that the incidence of ASDis requiring reoperation is higher. Zigler *et al*.^15^ believe that the incidence of ASDeg after ACDF is as high as 54.7% and the rate of ASDis reoperation is as high as 11.6%. Buttermann *et al*.^3^ found that 29% of patients with ASDis needed secondary surgery after 10 years of follow‐up after ACDF. Ahn *et al*.^1^ provided evidence that the anterior vertebral plate and ACDF increase the risk of ASDis and pointed out that this finding is consistent with the findings of randomized controlled trials conducted in the United States, in which the anterior vertebral plate fixation method and ACDF were used. In Europe and other countries, ACDF usually does not include the front side of the steel plate, and only the cages are involved. The original purpose of using a steel plate is to reduce the incidence of pseudarthrosis in the surgical segment, but a meta‐analysis reported by Shriver *et al*.^16^ showed that the risk of pseudarthrosis was low. Ji *et al*.^17^ reported that there was evidence that the use of the anterior vertebral plate fixation method, together with ACDF of the two segments, increases the incidence of ASDis compared to the use of an ACDF without the anterior vertebral plate method. In another analysis, Park *et al*.^18^ reviewed the lateral radiographs of 118 patients with anterior cervical fusion and found that the probability of adjacent disc degeneration increased in patients with a distance between the edge of the anterior vertebral plate and the adjacent disc of <5 mm. In a biomechanics study, Eck *et al*.^7^ reported that the use of prevertebral plates may accelerate the motion of adjacent segments, leading to ASDis. Our study results showed that ASDis was mostly located in the C_3/4_ segment and not in the gap below the surgical segment.

Matsumoto *et al*.^19^ found through imaging studies that the C_3/4_ and C_6/7_ intervertebral space heights of the non‐surgical segment in an anterior cervical fixation group indicated progressive spinal stenosis. Maiman *et al*.^20^ showed that the pressure of the C_4/5_ intervertebral disc after C_5/6_ internal fixation was significantly higher than that of C_5/6_ after C_4/5_ internal fixation; thus, it was considered that the pressure increase of the upper intervertebral disc of the fixed segment was more obvious than that of the lower disc. Chang *et al*.^4^ found the same result by studying cadaver specimens. The upper intervertebral space pressure was higher in the flexion/extension position. Chung *et al*.^5^ conducted a biomechanical study and reported that the upper articular surface pressure of the adjacent segment was higher than that of the normal non‐surgical group, and the pressure increased by 31.5%.

### Basis for Selecting SLAC as Secondary Surgery

ASDis was characterized by the presence of a screw and anterior plate in the vertebral body (Figs 1B,2B,3A,B). Even when the anterior plate and screw can be removed and reconstructed with ACDF, new screws still need to be inserted. As the bone of the lower vertebral body is destroyed by the last screw, the holding force of the new screws will be significantly reduced, and internal fixation will fail.

In a meta‐analysis article, although the JOA score and clinical symptom relief after ACDF were significantly better than after laminoplasty, the surgical technique and the rate of complications, such as cerebrospinal fluid leakage, internal fixation displacement, hematoma, and esophageal perforation^21^, ^22^, ^23^, ^24^, ^25^, ^26^, ^27^ were higher with ACDF. Injury to the recurrent laryngeal nerve, postoperative dysphagia, and hoarseness were common^2^, ^28^. Eichholz and Ryken^8^ analyzed 30 cases of patients with cervical revision surgery, and the complication rate was 27%. Hannallah *et al*.^29^ performed a statistical analysis of 1994 patients who underwent cervical spine surgery and found the incidence of postoperative cerebrospinal fluid leakage was 1% and the incidence of cerebrospinal fluid leakage during the revision surgery was 2.77 times that of the first surgery. Eichholz and Ryken^8^ believe that some implantable barriers administered during surgery cannot prevent the formation of scar tissue.

Therefore, when there is a requirement for a second surgery for ASDis, although ACDF can be performed again, some cases may require a multi‐segment ACDF, which makes the operation difficult because the anatomical level of scar tissue is unclear. Moreover, the technical skill level and operative experience of doctors in primary‐level hospitals may be limited. Using SLAC with indirect decompression, the risk is relatively low, the operation is simple, and the learning curve is low. Thus, the attending physician can complete the surgery independently.

For 52 patients, SLAC was performed the secondary surgery at our hospital, and these patients' clinical results were satisfactory.

### SLAC Requires Laminectomy and C_2_ Laminoplasty‐dome

In the 13 SLAC cases treated at our hospital, although the C_2/3_ and C_3/4_ segments of the intervertebral disc did not present with degeneration, protrusion, or spinal stenosis on imaging before the first operation, ASDis developed in 33 months (21–59 months) postoperatively. There were four (30.8%) cases of ASDis at C_2/3_ and eight (61.5%) cases of ASDis at C_3/4_. Increased contact and pressure during treatment or adjacent levels of facet joints may lead to micro‐damage of the facet joint and eventually accelerate the degradation of the adjacent segment facet joint^30^, ^31^.

Therefore, we recommend (especially for patients older than 70 years) that SLAC should include C_3_ laminectomy and C_2_ dome laminoplasty to avoid a second operation on C_2/3_ or C_3/4_ for ASDis, even if the intervertebral discs of the C_2/3_ and C_3/4_ segments do not show degeneration, protrusion, or spinal stenosis before the initial surgery and there are no corresponding symptoms. Because of the high risk and difficulty of reoperation, the following indications should be considered carefully:

1. Residual or progressive compression confirmed on imaging, positive conservative treatment for 3 months (excluding myofascial pain) with no real improvement in the symptoms and signs, and detrimental effects on the work and life of the patients.
2. There are two “threshold values” for selecting ACDF or SLAC. Fujimori *et al*.^32^, Denaro *et al*.^6^, and Kim *et al*.^33^ reported that when ossification of the posterior longitudinal ligament accounts for 50% of the cross‐sectional area of the spinal canal, laminoplasty is less effective in relieving neurological symptoms than ACDF. Suda *et al*.^34^ and Uchida *et al*.^35^ reported that when cervical kyphosis is more than 10°, the effect of laminoplasty in relieving neurological symptoms is inferior to that of ACDF. Therefore, when the patient meets the two abovementioned criteria, posterior cervical SLAC can be considered for patients who are older, have other diseases, or are unable to undergo anterior reoperation.
3. The social factors related to the patient, including mental status (presence of depression), age (presence of menopausal syndrome), marital emotional state, working status, economic status, and social identity must be considered. Patients often have high expectations and more negative emotions regarding revision surgery. Surgeons should communicate more with patients and their families, appropriately reduce the patient's expectations of the efficacy of the surgery, and consider social and legal issues, such as possible medical litigation and medical compensation.

## Limitations

This study had several limitations. First, the retrospective design introduced a degree of uncertainty because of missing and erroneous data in the medical records, as well as the lack of clinical information. Second, the small sample size likely affected the strength of the statistical analysis of the demographic and radiologic parameters. Third, the follow‐up period was relatively short.

## Conclusions

In conclusion, the main causes of ASDis include a distance of <5 mm between the anterior cervical plate and the adjacent intervertebral disc, and the screw breaking through the adjacent segment of the annulus. In this study, we found that ASDis developed most commonly at the C3/4 level. We recommend that during reoperation, when cervical kyphosis is <10° and the intraspinal protrusions account for <50% of the spinal canal cross‐sectional area, SLAC should be performed at the primary hospital. We suggest that this is a relatively simple technique, the local anatomy is clear, and the clinical results are satisfactory. Further studies with a larger sample size are required to corroborate our recommendations.
